# Spectrum of Clinical Presentation of Celiac Disease in Pediatric Population

**DOI:** 10.7759/cureus.15582

**Published:** 2021-06-10

**Authors:** Imran ., Huma Arshad Cheema, Muhammad Arshad Alvi, Mujeeb Ur Rehman, Muhammad Ali, Hussan Ali Sarwar

**Affiliations:** 1 Pediatric Gastroenterology, Hepatology and Nutrition, The Children's Hospital and The Institute of Child Health, Lahore, PAK; 2 Pediatric Cardiology, National Institute of Cardiovascular Diseases, Karachi, PAK; 3 Pediatric Gastroenterology, Hepatology and Nutrition, The Children’s Hospital and The Institute of Child Health, Lahore, PAK

**Keywords:** clinical spectrum, classic celiac disease, non classic celiac disease

## Abstract

Background

Classic form of celiac disease (CCD) presents with diarrhea and is traditionally taught as malabsorption syndrome. This form of CD is diagnosed with ease but non-classical form presenting without diarrhea is often missed and heavily underdiagnosed.

Objective

To determine the clinical spectrum of patients with CD.

Methods

This study was conducted in the Department of Gastroenterology & Hepatology at the Children’s Hospital, Lahore. Confirmed CD children according to NASPGHAN (North American Society of Pediatric Gastroenterology, Hepatology & Nutrition) criteria were enrolled in this study from June to September, 2020. Initial positivity followed by small bowel biopsy with Modified Marsh 2 and above is considered consistent with the diagnosis of CD.

Results

A total of 90 patients were selected according to NASPGHAN criteria, 77 (85.6%) patients had CCD whereas 13 (14.4%) patients had non-classical CD (NCCD). The mean ± SD age at diagnosis of CCD was 2.6 ± 2.3 years whereas mean ± SD in NCCD was 9 ± 1.8 years. Females clearly predominate in our cohort in general. Chronic diarrhea followed by failure to thrive (85%) were the most common symptoms in CCD whereas short stature (69%) was the most frequent feature in NCCD. Among CD patients, tissue transglutaminase-​immunoglobulin A (TTG-IgA) titre was significantly high (>10 times) in 80% of CD patients and the rest had positivity but not up to 10 times. There was no significant difference in titre of anti-TTG between CCD and NCCD.

Conclusion

Classical CD is still the most common in developing countries like Pakistan. High index of suspicion of CD should be maintained for patients who present with short stature, recurrent abdominal pain and refractory anemia.

## Introduction

Celiac disease (CD) is an immune-mediated systemic disorder elicited by gluten and related prolamines in genetically susceptible individuals resulting in injury to small intestinal mucosa [1]. The prevalence of CD is about 1% around the globe with female predominance [2]. Unfortunately, there are no epidemiological studies that show the actual prevalence of CD in the Pakistani population. 

CD occurs due to interplay between genetic and environmental factors [3]. Almost all people with CD carry HLA-DQ2 or HLA-DQ8. The gliadin peptide gains entry into the small bowel submucosa where it binds with HLA peptides on antigen-presenting cells leading to intraepithelial cell proliferation through activation of helper T cells [4]. This causes villous atrophy, crypt hyperplasia and the formation of antibodies related to CD.

CD has a wide spectrum of clinical presentation. The classic form with the intestinal symptoms predominates in infancy. In this pattern, chronic diarrhea, failure to thrive and abdominal distension are the predominant features but this represents the tip of the iceberg of the overall disease burden [5,6]. Significant number of patients with non-classical form are recently being described in the literature. They present in the form of failure to thrive, short stature, refractory anemia, unexplained rickets, vomiting, recurrent oral ulcers, recurrent abdominal pain and constipation [7-9]. We aimed to conduct this study to delineate the different presentations of celiac disease, especially the non-classical form in our populations. This will help us to diagnose the disease early and reduce short- and long-term morbidities. Moreover, this will open research gate in resource-limited countries for a better understanding of epidemiology of CD.

## Materials and methods

All the diagnosed cases of CD from June to September 2020 at the Department of Gastroenterology & Hepatology, The Children’s Hospital & Institute of Child Health, Lahore were studied. Children between nine months and 16 years of either gender diagnosed as CD based on NASPGHAN (North American Society of Pediatric Gastroenterology, Hepatology & Nutrition) criteria were included in this study [10]. All patients with raised anti-TTG (>18IU/L) underwent small bowel biopsy. Modified Marsh criteria were used to classify the biopsy findings. Marsh grade 2 and above were consistent with celiac disease. Chronic diarrhea was defined as diarrhea that lasts for more than 14 days [11]. Those patients in whom chronic diarrhea was the major phenotype were considered as classical CD (CCD).

Patients who did not present with chronic diarrhea and has other features of CD like short stature, anemia, abdominal pain were considered as non-classic or non-diarrheal phenotype. Short stature was defined as height for age less than -2 Z-score. Those patients were considered as failure to thrive whose weight for age fall below 3rd centile or weight deceleration that crosses two major percentile lines on growth chart [11]. 

Information regarding gender, clinical presentation, serological tests and small intestinal biopsy for CD were documented. Genetic testing was not done due to cost issues. Categorical data were summarized as numbers and percentages, whereas continuous data were summarized as mean and standard deviation. Comparison between groups for categorical variable was carried out using chi-square test, whereas for continuous variable t-test was used. The study was approved in ethical review board meeting of Children’s Hospital & Institute of Child Health.

## Results

There were a total of 90 children who met the inclusion criteria for CD. CCD was more common and comprised of 77 (85.6%) patients whereas 13 (14.4%) of patients were falling into non-classic category (0.000). The mean ± SD age at diagnosis of CCD was 2.6 ± 2.3 years whereas mean ± SD in NCCD was 9 ± 1.8 years (P = 0.001) having slight female predominance (P = 0.168). The maximum patients with CD were less than 10 years (<5 years (43/90, 47%), 5-10 years (32/90, 35%)) as shown in Table 1.

**Table 1:** Demographic features of celiac disease.

Variables	Number of patients	Percentage
Male	38 (n = 90)	42.7%
Female	51 (n = 90)	57.3%
Age less than five years	43 (n = 90)	49.4%
Six to 10 years	32 (n = 90)	36.8%
Greater than 10 years	12 (n = 90)	13.8%

Overall the most common symptoms in order of frequency are failure to thrive 76 (85%), chronic diarrhea 75 (84%), anemia 64 (84%), abdominal distension 67 (75%), short stature 51 (62%), recurrent abdominal pain 48 (54%), rickets 30 (34%), vomiting 23 (26%), constipation 7 (8%), recurrent oral ulcers 5 (6.7%) as given in Table 2.

**Table 2:** Clinical spectrum of celiac disease.

Symptoms	Percentage	Classic celiac	Non-classic celiac	P-values
Chronic diarrhea	84.4%	98%	0%	0.000
Abdominal distention	75.0%	69%	30.8%	0.000
Failure to thrive	85.6%	90%	45%	0.000
Short stature	62.9%	62%	69%	0.015
Refractory anemia	84.3%	82%	53%	0.000
Rickets	34.1%	38.7%	7.7%	0.003
Vomiting	26.7%	29.9%	7.7%	0.000
Recurrent oral ulcer	6.7%	5.3%	15.4%	0.000
Recurrent abdominal pain	53.9%	57.9%	31%	0.458
Constipation	7.8%	6.5%	15.4%	0.000

Most common symptoms in classic celiac disease in order of frequency are chronic diarrhea 75 (98%), failure to thrive 69 (90%), refractory anemia 63 (82%), abdominal distension 53 (69%). In non-classic type, short stature 9(69%), refractory anemia 7 (53%), FTT 6 (45%), abdominal pain 4 (31%). Significantly more percentage of CCD patients are failure to thrive as compare to NCCD (P = 0.000). Short stature was more common in non-classic variety (P = 0.015). In classic celiac, advanced grades of Marsh criteria were more common. In the non-classic type, 50% of biopsy findings were 3a and 50% were 3b. Type 1 diabetes mellitus was found in one patient, whereas one patient had association with epilepsy.

## Discussion

CD is commonly underdiagnosed due to variable presentation of the disease, especially the non-diarrheal variety. CD is classically a disease of gastrointestinal tract and chronic diarrhea is the predominant symptom.

Though our study showed 14% of patients with non-classic type of celiac disease. Recent studies are reporting the increasing prevalence of non-classical variety. Like the one performed by Farhan et al that showed 39.97% prevalence [12]. This may in part be related to under diagnosis of non-celiac disease in our community that may be related to high threshold of physicians for investigating patients with non-classic presentations of CD. The mean age at diagnosis of CCD is 5.6 years which is similar to the study Aziz et al [13]. The early presentation of CCD and late presentation of NCCD is documented in many studies. In all cases of CD failure to thrive was the most common clinical presentation (85%), followed by chronic diarrhea (84%), anemia (84%). These findings are consistent with Kliegmann and Jenson in which failure to thrive is the most common presentation (85.7%) followed by diarrhea (71%) [11]. One reason behind this is that failure to thrive is related to both classic and non-classic types of celiac disease. Patients with failure to thrive were more common in classic type (92%). In non-classic type, it is 46%. Anti-TTG IgA was 10 times raised in 80% of patients. Anti-TTG IgG was more than 10 times in 44%. In terms of Anti-TTG IgA both classic and non-classic types have an equal percentage of significantly positive and negative values. Most of our patients in CD fall in Marsh 3b. Marsh 3b and advance stage were more commonly present in classic celiac disease as compare to non-classic variety, in which early grade was more common. This is in accordance with multiple studies that show a high grade of histopathological findings in patients with gastrointestinal symptoms [13]. Although villous atrophy is the hallmark of CD. There are two patients in our study that fall in Marsh 1 category but their presentation as well as serology favor the diagnosis of CD. Also their clinical condition improved on gluten-free diet. Small intestinal lesion has a spectrum of severity, moreover the lesion is characteristically patchy and potentially can be missed on biopsy. Therefore in symptomatic children with positive serology, the diagnosis of CD can be entertained even in the absence of favoring histology. It is also important to note that small bowel histology may improve significantly if the patient reduces or eliminates gluten from his diet. Therefore patients with suspected celiac disease should be instructed to take gluten-containing diet until small bowel biopsy is done [14]. Short stature is the most common extraintestinal manifestation of NCCD found in 69% of our patients. Short stature can be the only presentation of celiac disease. In our study, 30% of NCCD presented with short stature only. This is quite similar to the study performed by Aziz et al [13]. Therefore many studies recommend screening for CD in severely short stature. Anemia is more commonly reported in CD throughout the globe and it has been observed that low hemoglobin may not improve even after one year of gluten-free diet [13]. In our study 84% of patients had anemia. This is quite similar to the study performed by Poddar et al that showed 84% of patients with anemia in celiac disease [15].

In our study, 1% of patients had insulin-dependent diabetes mellitus. Various studies have shown 1%-19% prevalence of celiac disease type 1 diabetes mellitus [16].

## Conclusions

Though CCD is still the most common presentation of celiac disease in our set up, there is some under diagnosis of CD, especially patients with NCCD. So one should have high index of suspicion to patients with short stature, recurrent abdominal pain, recurrent oral ulcers, refractory anemia. They should be investigated for celiac disease. Comorbid conditions and associations may arise during the course of CD and they need to be followed in outdoor clinics.
